# Evaluation of an in-clinic Serum Amyloid A (SAA) assay and assessment of the effects of storage on SAA samples

**DOI:** 10.1186/1751-0147-52-8

**Published:** 2010-02-02

**Authors:** Anna Hillström, Harold Tvedten, Inger Lilliehöök

**Affiliations:** 1 University Veterinary Hospital, Swedish University of Agricultural Sciences, 750 07 Uppsala, Sweden; 2 Strömsholm Referral Animal Hospital, 734 94 Strömsholm, Sweden; 3 Department of Clinical Sciences, Swedish University of Agricultural Sciences, 750 07 Uppsala, Sweden

## Abstract

**Background:**

An in-clinic assay for equine serum amyloid A (SAA) analysis, Equinostic EVA1, was evaluated for use in a clinical setting. Stability of SAA in serum samples was determined.

**Methods:**

Intra- and inter- assay variation of the in-clinic method was determined. The in-clinic method (EVA1) results were compared to a reference method (Eiken LZ SAA) with 62 patient samples. For samples with SAA concentrations within the assay range of EVA1 (10-270 mg/L), differences between the methods were evaluated in a difference plot. Linearity under dilution was evaluated in two samples. Stability of SAA in three serum pools stored at 4°C and approximately 22°C was evaluated with the reference method day 0, 1, 2, 4, 7, 17 and analysed with a two-way ANOVA.

**Results:**

The imprecision (coefficient of variation, CV) for the in-clinic method was acceptable at higher SAA concentrations with CV values of 7,3-12%, but poor at low SAA concentrations with CV values of 27% and 37% for intra- and inter-assay variation respectively. Recovery after dilution was 50-138%. The in-clinic assay and the reference method identified equally well horses with low (<10 mg/L) and high (>270 mg/L) SAA concentrations. Within the assay range of the in-clinic method, 10-270 mg/L, the difference between the two methods was slightly higher than could be explained by the inherent imprecision of the assays. There were no significant changes of serum SAA concentrations during storage.

**Conclusions:**

The in-clinic assay identified horses with SAA concentrations of <10 mg/L and >270 mg/L in a similar way as the reference method, and provided an estimate of the SAA concentration in the range of 10-270 mg/L. The imprecision of the in-clinic method was acceptable at high SAA concentrations but not at low concentrations. Dilution of samples gave inconsistent results. SAA was stable both at room temperature and refrigerated, and thus samples may be stored before analysis with the reference method.

## Background

Increased concentration of serum amyloid A (SAA), a major acute phase protein, is the most sensitive method to identify inflammation in horses. Healthy horses have very low SAA concentrations, whereas there is a rapid and large increase of SAA following inflammatory stimulus [1-3]. Other tests of inflammation such as total leukocyte counts, differential leukocyte counts and fibrinogen concentration have been more readily available to equine practitioners, but are rather insensitive indicators of inflammatory diseases in horses [4-6]. SAA rises fast after an inflammatory stimulus and, due to its short half-life in serum [7], the concentration diminishes rapidly during recovery. SAA is a non-specific inflammatory marker and increased concentrations are seen in bacterial infections [3,8-10], viral infections [1,11] and in sterile inflammation [6]. The increase in SAA concentration has been shown to correlate with severity of clinical signs [11] and with the intensity of surgical trauma [12]. However, there is also individual variation as a similar experimentally induced trauma elicited varying responses of SAA among individuals [3].

Methods for determining equine SAA have been cumbersome to perform or require automated analysers. These assays include electroimmunoassay [1], single radial immunodiffusion [2], slide reversed passive latex agglutination [13], non-competitive chemiluminescence enzyme immunoassay [3], ELISA [14] and assays using the latex agglutination immunoturbidometric principle [15,16]. An in-clinic test intended for measuring equine SAA, Equinostic EVA1 (Equinostic ApS, Birkerød, Denmark), has become available and was reported to have good performance in a laboratory setting [17]. In this study the method was evaluated in a clinical setting at a private veterinary hospital. Additionally the stability of SAA in serum, analysed with an immunoturbidometric method used at a referral laboratory, was investigated.

## Methods

### SAA analyses

The in-clinic method EVA1 is an immunoturbidometric assay where a reaction between SAA and anti-SAA-antibodies causes an increased turbidity, which is measured with a spectrophotometer. Analysis with the in-clinic method EVA1 (lot 78005) was performed according to the manufacturer's instructions as previously described [17], at a regional equine hospital (Strömsholm Referral Animal Hospital, Strömsholm, Sweden). The sample volume of 2 μl serum was measured with a capillary tube. Written information about five standard points and their respective absorbance was enclosed with each lot of reagent, and a calibration curve was created by transferring these data to the EVA1 instrument. The manufacturer reported an assay range of approximately 10-300 mg/L, but 270 mg/L was set as the upper limit in this study, because this was equal to the concentration of the highest standard point in the actual lot. An equine serum pool was used as control and was analysed once every week.

The reference method Eiken (LZ test SAA, Eiken Chemical Co Tokyo, Japan) is a human immunoturbidometric assay previously validated in horses [15]. Analyses with the reference method were performed at the Clinical Pathology Laboratory at the University Animal Hospital (Swedish University of Agricultural Sciences, Uppsala, Sweden) according to the manufacturer's instructions with an automated analyser, Konelab (Konelab PRIME 30, Thermo Fisher Scientific Inc, Waltham, USA). Samples with SAA concentrations of >250 mg/L were diluted 1:6 with distilled water on the automated analyser. An equine serum pool and a human commercial control sample (Trulab P, Diagnostic Systems International, Holzheim, Germany) were used as controls and were analysed daily. Inter-assay variation of the reference method was approximately 7.5% (data not shown).

### Animals and samples

Intra- and inter-assay variations were determined with two serum pools prepared by mixing sera from 5 equine patients. Four of the 5 horses had elevated SAA concentrations and 1 had SAA concentration of < 5 mg/L. In a comparison study blood samples from 62 adult equine patients at Strömsholm Referral Animal Hospital were collected from the jugular vein into serum clot tubes. The selection criterion was that the clinician had also requested plasma fibrinogen. Samples were centrifuged at 1000 g for 10 minutes after clotting was completed. For the stability study three serum pools (pool A-C) were prepared from sera from 6 different horses, 5 of which had elevated SAA concentrations and 1 that had SAA concentration of < 5 mg/L.

### Characteristics of in-clinic assay (EVA1)

Intra-assay variation was determined by analyzing two serum pools with SAA concentrations of 12 mg/L and 197 mg/L respectively, ten consecutive times. The coefficients of variation (CV) in % were calculated (standard deviation divided by mean value × 100) to describe imprecision. Inter-assay variation was determined by analyzing, on 10 different days within a period of 30 days, the two serum pools mentioned above which were frozen in aliquots at -20°C until analysis. Only the vial needed for each analytical run was thawed. Intra-assay variation was also measured by analysing a commercial control sample from Equinostic, with a stated mean value of 150 mg/L, ten times in a row.

Inaccuracy was evaluated by diluting two samples with SAA concentrations of 212 mg/L and 255 mg/L 1:2, 1:4 and 1:8 with PBS, a buffer solution (0.15 M, pH 7.4) provided by the manufacturer.

### Comparison between in-clinic method (EVA1) and reference method (Eiken)

All samples in the comparison study were analysed with the in-clinic assay at Strömsholm within 5 hours of collection. These samples were handled as clinical samples, which meant they were analyzed among other patient samples in order that they arrived to the laboratory unless labelled as acute. One laboratory technician performed all the analyses on the in-clinic assay. Samples were then transported to the University laboratory for analysis with the reference method, Eiken. Forty-nine of the samples were analysed within 24 hours, 6 samples within 2 days and 7 samples within 3-5 days. Samples were continuously stored at 4°C.

### Storage stability of SAA in serum

Stability of SAA in serum was investigated with three serum pools (pool A, B and C) with SAA concentrations of 1060, 197 and 73 mg/L, respectively. The serum pools were stored at both 4°C and at room temperature (approximately 22°C). They were analysed with the reference method on day 0, 1, 2, 4, 7 and 17.

### Statistical analyses

Arithmetic means, standard deviations and intra- and inter-assay CV were calculated using descriptive statistical procedures (Microsoft Excel 2003, Redmond, USA). The differences between the in-clinic and the reference method in the comparison study were compared with the limits of inherent imprecision in a difference plot [18] (Analyze-it Software Ltd, Leeds, UK). The combined imprecision was calculated by using the inter-assay variations of EVA1 and Eiken (CV 12% and 7.5% respectively) in the formula √(CV^2^_EVA1 _+ CV^2^_Eiken_). Storage stability was evaluated by a 2-way ANOVA with temperature (4°C and 22°C) and time (day 1, 2, 4, 7 and 17) as factors. A significance value of < 0.05 was used.

## Results

### Characteristics of in-clinic assay (EVA1)

Results from the precision study are shown in table 1 and results from the dilution study in table 2.

**Table 1:** Intra- and inter-assay variation in determining SAA with EVA1

	No. of runs	SAA concentration (mg/L)		Coefficient of variation (%)
		Mean	SD	
**Intra-assay**	10^a^	12	3.3	27
	
**EVA1**	10^b^	197	14	7.3
	
	10^c^	159	16	9.9

**Inter-assay**	10^a^	22	8.3	37
	
**EVA1**	10^b^	171	20	12

^a^Serum pool with low SAA concentration.

^b^Serum pool with high SAA concentration.

^c^Control sample from Equinostic with a stated mean value of 150 mg/L.

SD; standard deviation

**Table 2:** Dilution of two SAA samples

	**SAA (mg/L)**	**SAA calc** **(mg/L)**	**Recovery (%)**	**SAA (mg/L)**	**SAA calc** **(mg/L)**	**Recovery (%)**
	
**Undiluted**	212	NA	NA	255	NA	NA
	
**Diluted 1:2**	103	106	97	176	128	138
	
**Diluted 1:4**	41	53	78	73	64	115
	
**Diluted 1:8**	<10	27	-	16	32	50

NA = not applicable

SAA calc = SAA calculated

### Comparison between in-clinic method (EVA1) and reference method (Eiken)

The assay range of EVA1 was 10-270 mg/L. In the comparison study low SAA concentrations of < 10 mg/L were found in 19 samples (31%) with both the in-clinic and the reference method. One sample had a SAA concentration of <10 mg/L with the in-clinic method and 19 mg/L with the reference method. Thirty one samples (50%) had SAA concentrations of more than 270 mg/L with both assays. The maximum assay range of EVA1 was 270 mg/L and therefore dilution of samples would have been necessary to obtain results greater than 270 mg/L, but were not performed in the present study.

**Figure 1:**
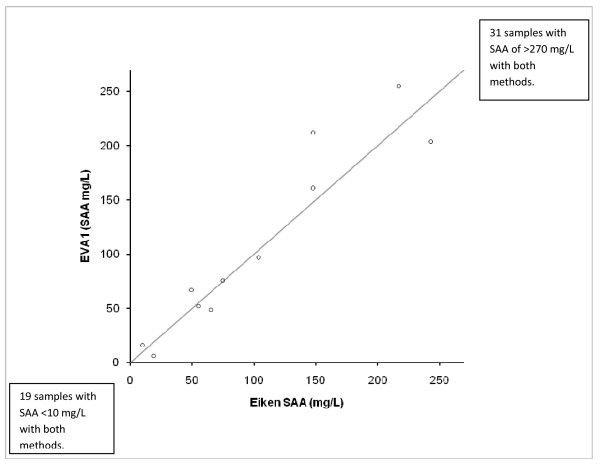
**Method comparison between in-clinic assay (EVA1) and reference assay (Eiken)**. Eleven samples with SAA concentrations of 10-270 mg/L (assay range of EVA1) are plotted. The line represents y = x. One sample had a SAA concentration of <10 mg/L with the in-clinic method and 19 mg/L with the reference method (not shown in this figure). Nineteen samples had SAA concentrations of <10 mg/L with both methods and 31 samples had SAA concentrations of >270 mg/L with both methods.

**Figure 2:**
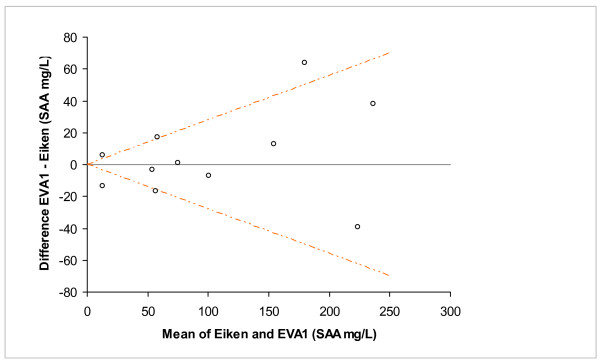
**Difference plot of SAA concentrations measured by in-clinic method (EVA1) and reference method (Eiken)**. The dashed lines represent the 95% confidence interval of the combined inherent imprecision of the two assays.

Only 11 samples (18%) had SAA concentrations of 10-270 mg/L with the in-clinic method (Figure 1 and 2). The combined imprecision was 14%, calculated by using the inter-assay variations of both methods. In figure 2 the dashed lines represent the 95% confidence interval of this combined inherent imprecision [18]. Because 5 of the 11 samples were outside the interval outlined by the lines, the difference between the two methods was larger than could be explained by imprecision only.

### Storage stability of SAA in serum

**Figure 3:**
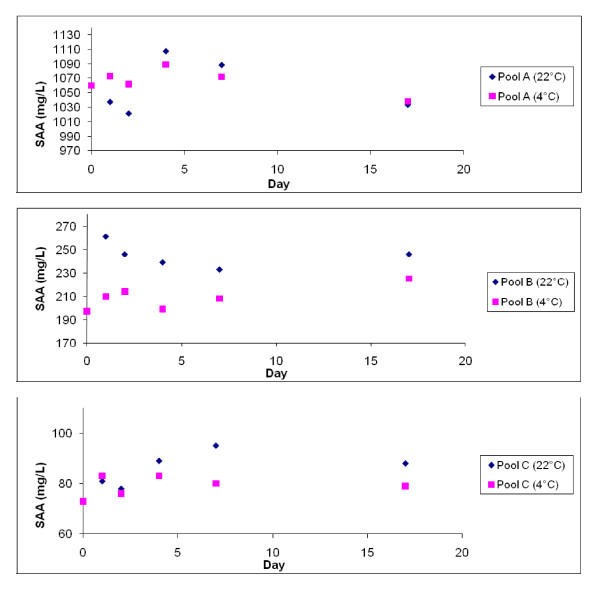
**Storage stability of SAA in three serum pools stored at 4°C and approximately 22°C**.

There were no statistical significant changes in SAA concentrations over time in 3 serum pools stored at 4°C and 22°C (Figure 3), and the variance between days was not higher than could be explained by the imprecision of the method. In pool B there was a significant difference in SAA concentrations between the pool stored at 4°C and 22°C (p = 0.003).

## Discussion

The Equinostic EVA1 in-clinic instrument was as effective as the reference method in identification of horses with SAA concentrations of < 10 mg/L or > 270 mg/L. Fifty one of 62 horses in this study belonged to one of these groups. Only 11 horses had SAA concentrations of 10-270 mg/L, which reflects the biological behaviour of SAA since the elevation of SAA in inflammation is often more than 100-fold [15]. The difference between the two methods in the range of 10-270 mg/L was slightly larger than could be explained by the inherent imprecision of the assays, but the clinical interpretation would be mild-moderate elevations with both the in-clinic and the reference method. In the previous validation study the in-clinic method constantly underestimated the SAA concentration compared to the reference method [17], but this bias was not seen in the present study including only 11 samples within this range.

The imprecision of EVA1 was higher than has been reported earlier [17]. A possible explanation for this, and for the differences in the method comparison study, may be that in this study the analyses were handled as routine samples in the daily work at the laboratory, and not as a research project. The person analysing the samples worked routinely in an acute type laboratory with a high volume of samples, with many samples expected to be analysed within 30 minutes. The person performing the tests was a trained medical technician, working in a higher quality laboratory with only professional and experienced personnel. Even worse performance might be expected in a clinic without licensed laboratory professionals where laboratory testing is done by the person available at the time. Some manipulations in the EVA1 method require good laboratory technique and provide risk of error with inexperienced operators. For example, the sample volume of 2 μl is so small that variation in this volume among untrained operators is likely.

At high SAA concentrations the CV of the in-clinic method for inter-assay variation was 12%, which is acceptable for clinical purposes considering the biological behaviour of SAA. At low SAA concentrations the precision was poor with a mean CV of 32%. It is recommended that each laboratory using the in-clinic method evaluate their own imprecision at different concentrations, and also determine reference values and cut off values. The manufacturer suggested a reference value of < 25 mg/L. The EVA1 instrument reported SAA concentrations out to two decimal places, however, given the imprecision of the method, should rather be whole numbers.

The in-clinic method was previously reported to have an acceptable linearity under dilution [17]. In our study, the recovery varied considerably (50-138%). It is not clear whether this was due to manual errors or if there was a problem with the assay. Results were poorest at low SAA concentrations, and in this range the high imprecision could contribute to the inaccurate results. Dilution of samples would be required to be able to determine SAA concentrations of > 270 mg/L, and further investigations of effects of dilutions and accuracy with the in-clinic assay in a clinical setting would be required to validate the method for this purpose. The procedure of diluting a test doubles the reagent costs and more than doubles the time required for the SAA analysis.

In this study SAA was stable in both room temperature and at 4°C. A previous study of stability of SAA in equine samples stored at room temperature reported decreased levels of SAA after storage in 3 samples, whereas SAA concentrations in 10 other samples changed very little [19]. The reasons why the stability varied between samples are unknown. In another study where an equine SAA standard pool was stored at 4°C over a period of up to two months, no significant changes in SAA concentrations were noted [1]. The CV for inter-assay variation of the reference method (Eiken) was approximately 7.5% at the current laboratory, and in the stability study the variance was not higher than could be explained by this imprecision. Changes in SAA concentrations over time would possibly be discovered if using a more precise method, but would not likely be of clinical importance. In pool B there was a significant difference between SAA concentrations when stored at 4°C and 22°C respectively, but no similar trend was seen in the two other serum pools.

In the comparison study samples were analysed on the in-clinic assay on the day of collection and then stored up to 5 days at 4°C before analyse on the reference method. Ideally samples in method comparisons should be handled identically, but considering that the storage stability of SAA was satisfactory using the reference method, the storage times used in this study should not affect the results. However, as different antibodies were used in the two assays it cannot be concluded that the same stability would be present when analysing SAA with the in-clinic method. It is possible that some antibodies detect degraded SAA protein, while others do not. The stability of SAA with the in-clinic method was not evaluated. As the purpose of the in-clinic method is to measure SAA without delay, stability of SAA is less crucial for this assay than for the reference assay, where samples often need to be transported to a referral laboratory.

## Conclusions

The in-clinic method was effective in identifying horses with low (< 10 mg/L) or elevated (>270 mg/L) SAA concentrations and it provided an estimate of the SAA concentration in the range of 10-270 mg/L. The imprecision of the in-clinic method was acceptable at high SAA concentrations but not at low concentrations. Dilution of samples gave inconsistent results. SAA was stable for at least 17 days at room temperature and refrigerated, and thus samples may be stored before analysis with the reference method.

## Competing interests

The authors declare that they have no competing interests.

## Authors' contributions

All authors contributed in the planning of the study and writing of the draft. All authors read and approved the final manuscript.
